# The Importance of Exosite Interactions for Substrate Cleavage by Human Thrombin

**DOI:** 10.1371/journal.pone.0129511

**Published:** 2015-06-25

**Authors:** Gurdeep Chahal, Michael Thorpe, Lars Hellman

**Affiliations:** Department of Cell and Molecular Biology, Uppsala University, BMC, Box 596, SE-751 24, Uppsala, Sweden; Nagoya University, JAPAN

## Abstract

Thrombin is a serine protease of the chymotrypsin family that acts both as a procoagulant and as an anticoagulant by cleaving either factor VIII, factor V and fibrinogen or protein C, respectively. Numerous previous studies have shown that electropositive regions at a distance from the active site, so called exosites, are of major importance for the cleavage by human thrombin. Upstream of all the known major cleavage sites for thrombin in factor VIII, factor V and fibrinogen are clusters of negatively charged amino acids. To study the importance of these sites for the interaction with the exosites and thereby the cleavage by thrombin, we have developed a new type of recombinant substrate. We have compared the cleavage rate of the minimal cleavage site, involving only 8-9 amino acids (typically the P4-P4’ positions) surrounding the cleavage site, with the substrates also containing the negatively charged regions upstream of the cleavage sites. The results showed that addition of these regions enhanced the cleavage rate by more than fifty fold. However, the enhancement was highly dependent on the sequence of the actual cleavage site. A minimal site that showed poor activity by itself could be cleaved as efficiently as an optimal cleavage site when presented together with these negatively charged regions. Whereas sites conforming closely to the optimal site were only minimally enhanced by the addition of these regions. The possibility to mimic this interaction for the sites in factor V and factor VIII by recombinant substrates, which do not have the same folding as the full size target, indicates that the enhancement was primarily dependent on a relatively simple electrostatic interaction. However, the situation was very different for fibrinogen and protein C where other factors than only charge is of major importance.

## Introduction

**Fig 1:**
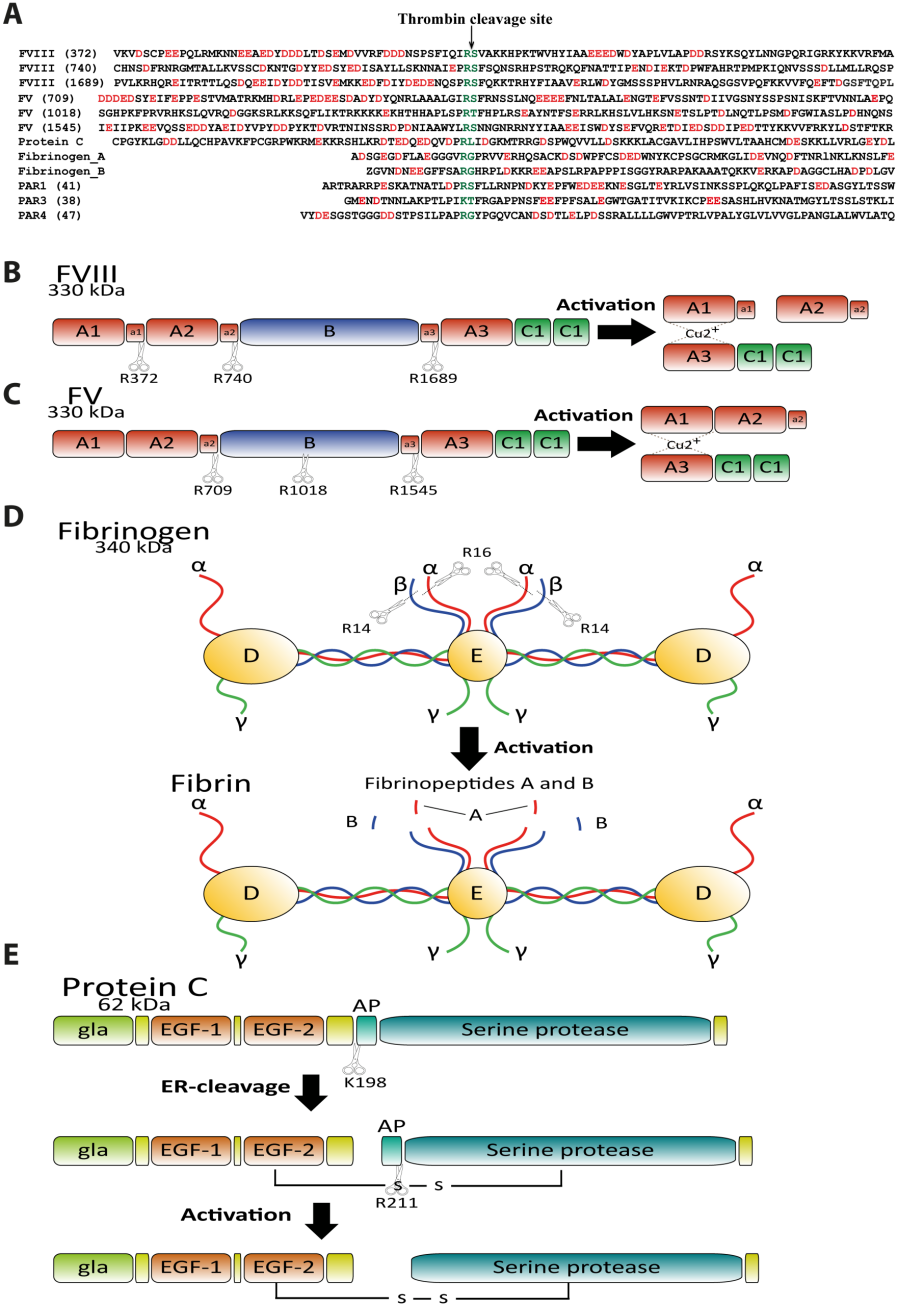
Regions surrounding cleavage sites for thrombin in a panel of important target molecules. Panel A shows the regions flanking the activating sites in natural substrates of thrombin. The amino acid sequences flanking both N-terminally and C-terminally of the cleavage sites for thrombin in FV, FVIII, PAR1,3 and 4, α & β fibrinogen and Protein C are depicted. Sequences are shown in a one-letter code and cleavage site numbering is based on the mature protein without signal peptide. The negatively charged amino acids are marked in red and thrombin cleavage sites are shown by arrows. Panels B-E shows schematic figures of FVIII, FV, fibrinogen and protein C showing the cleavage sites for thrombin (depicted by scissors with numbered residue).

**Fig 2:**
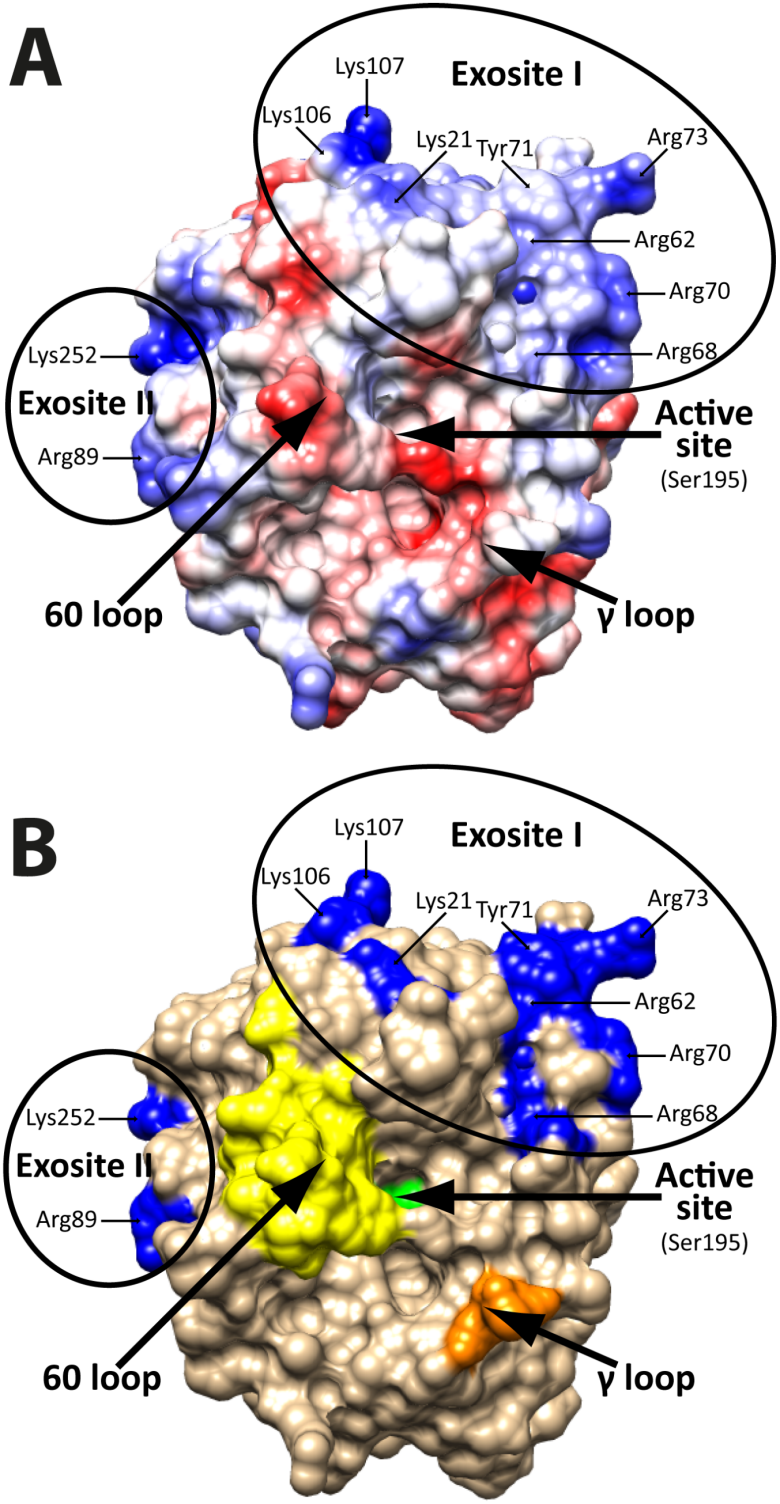
Schematic 3-D models of human thrombin. Panel A shows a schematic 3D structure of human thrombin with the charge density illustrated with blue for basic or positively charged regions and red for negatively charged or acidic regions. The color intensity reflects the charge intensity. Panel B shows a schematic 3D structure of human thrombin where some of the positively charged amino acid positions studied by site directed mutagenesis in exosites I and II are marked in blue. The active site serine is shown in green and the histidine and aspartic acids are hidden under an extending loop and therefore not visible in this angle of the molecule. Two extended surface loops, the 60 loop and the γ-loop are shown in yellow and orange, respectively. Thrombin structure from PDB, code 1DM4 run using UCSF Chimera v1.8 and annotated in Adobe Illustrator CS5.

Thrombin is a multifunctional serine protease belonging to the chymotrysin family and has a wide range of diverse biological functions. This enzyme has been the focus of intense study since its discovery in the 19th century [1]. It is the central bioregulatory enzyme in hemostasis with both procoagulant and/or anticoagulant activities. It is known to cleave a number of physiologically very important substrates including, fibrinogen, coagulation factor (F) V and FVIII as well as several of the protease activated receptors (PARs) and protein C (Fig 1) [2–11]. Human prothrombin, or factor II, is synthesized in the liver as a single polypeptide of 622 amino acids and secreted as a 72 kDa protein with four domains, an N-terminal γ-carboxyglutamic acid (GLA) domain, two kringle domains, and a serine protease domain [1]. After processing the active enzyme lacks its N-terminal domains and consists of only the serine protease domain containing two polypeptide chains A (36 residues) and B (259 residues), covalently linked through a disulfide bond [1]. The active site is formed by extending loops, the 60-loop above and the γ-loop below, meaning the active site is unusually deep for a serine protease of the chymotrypsin family (Fig 2) [1]. In addition to the residues within its active site, the specificity of thrombin for different substrates and inhibitors is determined by the two electropositive exosites, termed anion-binding exosites (ABEs)-I and-II (Fig 2) [1] [12, 13]. These sites have been named ABEs due to their apparent affinity for negatively charged ligands. ABE-I is present adjacent to the P’ side of the active site cleft and contains residues Arg20, Lys21, Arg68, Arg70, Arg73, Lys77, Lys106, Lys107, Lys154 whereas ABE-II contains residues Arg89, Arg93, Arg98, Arg123, Arg170, Lys174, Arg178, Arg245, Lys247, Lys248 and Lys 252 (Fig 2) [14]. The exosite interactions enable the formation or stabilization of the initial thrombin-substrate complexes sufficiently so that the peptide bond can be cleaved. Thrombin utilizes either or both of the two electropositive exosites to interact with its macromolecular substrates and cofactors. ABE-I has been shown to bind fibrinogen and the potent thrombin inhibitor hirudin plus multiple other proteins whereas ABE-II seems to primarily bind heparin [12, 13, 15].

The substrates we have chosen to study the role of exosite interactions are three sites within FV, three in FVIII, one in the fibrinogen α chain, one in the fibrinogen β chain and one site in protein C. These represent the majority of known important activation sites for thrombin and thereby give a good view of the factors that determine the importance of exosite interactions for this medically important enzyme.

One of these targets, coagulation FVIII is synthesized as a very large glycoprotein in the liver as a single chain polypeptide molecule of 2351 amino acids. After signal sequence removal and glycosylation, the protein has a molecular mass ≈ 300-kDa (Fig 1) [16, 17]. It circulates in plasma bound to von Willebrand factor (vWf) [18]. FVIII contains the following domains A1-A2-B-A3-C1-C2, and is activated through limited proteolysis by thrombin at three sites Arg372, Arg740 and Arg1689. Cleavage at Arg372 at the junction of the A1-A2 domains exposes the FIXa binding site. Cleavage at Arg740 at the A2-B domain border results in the release of the B domain, and cleavage at Arg1689 releases the acidic fragment A3 and leads to dissociation from its carrier protein, vWf, allowing FVIIIa to bind to phospholipid membranes (Fig 1) [19]. After proteolytic processing by thrombin, the active FVIII (FVIIIa) is a heterotrimer comprising of the A1, A2 and A3-C1-C2 subunits lacking the B domain [16]. FVIIIa serves as a cofactor for the activity of the serine protease FIXa in the intrinsic tenase complex on the surface of anionic phospholipids. Its activation by thrombin thereby up-regulates thrombin generation through a feedback mechanism [20].

The second target under analysis is human FV, which is synthesized in the liver as a single-chain glycoprotein of 2190 amino acids and is characterized by a domain structure A1–A2–B–A3–C1–C2 (Fig 1). FV circulates in the blood as a full-length protein and is activated by thrombin, releasing the B-domain by cleavage at residues Arg709, Arg1018, and Arg1545. Activated FV (FVa) is composed of the heavy (A1-A2 domains, 105 kDa) and light (A3-C1-C2 domains, 74 kDa) chains that are bound by a copper ion [3, 5, 21]. FVa acts as an essential cofactor in thrombin generation and the rate of the prothrombin to thrombin conversion by FXa is enhanced by several orders of magnitude in the presence of FVa and Ca2+ on phospholipid membranes [22]. The domain structure of FV is similar to that of the highly homologous FVIII (Fig 1).

The third target, fibrinogen, is a large complex of several polypeptides, the α chain, the β chain and the γ chain, where the activation involves the cleavage of the N-terminal of both the α and the β chains thereby exposing a site that facilitates the polymerization of the resulting fibrin molecules (Fig 1) [2]. The cleavage site of the α chain is in position 16 of the mature peptide and position 14 in the β chain (following signal sequence cleavage).

The fourth target, human protein C, is synthesized as a 461 amino acid polypeptide, which after signal cleavage at residue 18, is 442 amino acids in length [23]. After a second cleavage at a tribasic site at position 42, the protein is 419 amino acids in size [23]. The protein consists of four domains, an N-terminal γ-carboglutamic acid domain, two EGF domains and a C-terminal serine protease domain [24]. A third cleavage occurs by a yet unknown protease during ER transport at sites 198 and 199 removing a dipeptide [25]. The resulting two-chain protein C is connected by a disulphide bond between Cys183 and Cys319 (Fig 1) [25]. Activation by thrombin then removes a 12 amino acid long propeptide by cleavage at Arg211 (Fig 1) [25].

**Fig 3:**
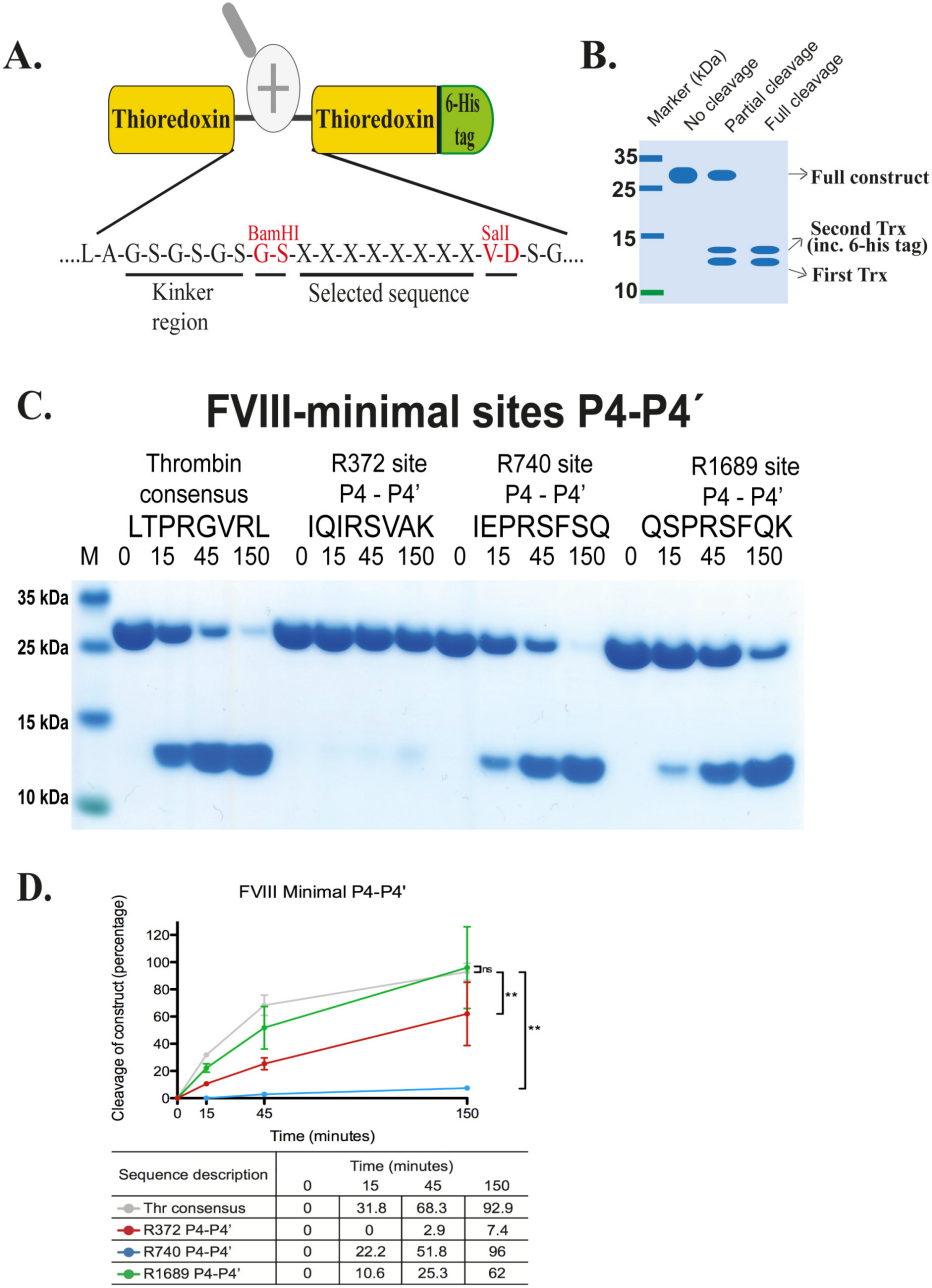
Analyses of the minimal sites for three thrombin cleavage sites in FVIII by the use of recombinant protein substrates. Panel A shows the overall structure of the recombinant protein substrates used for analysis. In these substrates, two thioredoxin (trx) molecules are positioned in tandem and the proteins have a His_6_-tag positioned in their C termini. The different cleavable sequences are inserted in the linker region between the two trx molecules with the use of two unique restriction sites, one *BamHI* and one *SalI* site, which are indicated in the bottom of panel A. Panels B shows a schematic representation of a cleavage reaction. The uncleaved substrates have a molecular weight of approximately 25 kDa and the cleaved substrates appear as two closely located bands with a size of 12–13 kDa. Panel C shows a comparative analysis of the cleavage efficiency of the thrombin consensus sequence with the P4-P4’ sequences from three cleavage sites in FVIII. The name and sequence of the different substrates are indicated above the pictures of the gels. The time of cleavage in minutes is also indicated above their corresponding lanes. In panel D we show an SDS-PAGE density summary of cleaved substrates. All protein gels were analyzed using Image Quant TL 1D gel density software (v8.1) from GE Life Science (Piscataway, NJ USA) or the UN-SCAN-IT Gel Analysis Software from Silk Scientific Inc. (Orem, Utah USA). Individual bands from the full-length constructs (top bands) were analyzed with manual lane editing and minimum profile. Bands were detected automatically and gating adjusted to compare cleavage over the time course. Figures show percentage cleavage of the original construct (time point 0 minutes). Standard deviation of the time points are shown (mean +- standard deviation). Statistical analyses were performed using the Mann-Whitney test with two-tailed P value.

A cluster of acidic residues localizes N-terminally, and sometimes both N- and C-terminally, of the cleavage sites for thrombin in FVIII, FV and fibrinogen α chain. These regions could potentially form an electrostatic complementarity to the electropositive exosites in thrombin for efficient cleavage (Fig 1). Even though both electropositive exosites within thrombin have been reported to be involved in the thrombin-catalyzed activation of FVIII, little is known about the mechanism and importance of these electronegative sites of the substrate [26, 27]. To determine the contributions of these negatively charged residues to the thrombin-catalyzed activation, we have used a new type of recombinant substrate recently developed in our lab [28, 29]. These substrates contain two bacterial proteins with a short linker in between, followed by a six-residue long histidine tag for purification purposes (Fig 3). In the linker region, a cleavable sequence is inserted. This region can accomodate sequences from a few amino acids long to potentially hundreds. The longest included so far is 94 amino acids, which is used for one of the FVIII sequences. These substrates can enable a quantitative analysis of the contribution of the negatively charged exosite interacting regions for the cleavage by thrombin, which had previously only been possible with long synthetic peptides or in some cases the entire target protein. Chromogenic substrates are usually short, 4–5 amino acid long peptides which are often used to study kinetics and cleavage specificity. However here they are not useful as they do not cover the entire cleavable or exosite-interacting regions, and therefore do not accurately reflect the situation endopeptidases encounter. This is especially true for complex enzymes such as thrombin that are highly dependent on their extended specificity.

In this study, the analysis of the importance of exosite interactions has been performed by comparing the cleavage efficiency of thrombin in two ways. Firstly, we compared the cleavage efficiency of the minimal cleavage sites P4-P4’ for each of the three proteolytic sites to an optimal site for thrombin (P4-P4’: LTPR↓GVRL) identified previously in our lab using phage display technology [28]. Furthermore, we compared the cleavage rates of these minimal sites with the cleavage sites including the N-terminal acidic region of 30–40 amino acids, as well as with mutated variants of this sequence, where approximately 50% of the negatively charged amino acids had been replaced with serine or glycine. We subsequently tested the combination of both N- and C-terminal regions for the possible additional enhancement of the C-terminal region on the cleavage efficiency. The size of the interacting regions could therefore be almost 100 amino acids long (90 and 94 amino acids were the two largest sequences). Similar analyses were then performed for three cleavage sites for thrombin in human FV, one in fibrinogen α chain, one in the fibrinogen β chain and one site in protein C. Using the recombinant substrates we have been able to obtain quantitative data on the involvement of these exosite interactions.

## Material and Methods

### Thrombin

Lyophilized powder of human activated plasma thrombin (Sigma T-6884) was diluted in double-distilled water to a concentration of 0.2 NIH units/ul. 0.2 U of diluted thrombin was used for cleavages of the various recombinant substrates.

### Generation of recombinant substrates for the analysis of the cleavage specificity

Two copies of the *E*.*coli* thioredoxin (trx) gene were inserted in tandem into the plasmid pET21-trx vector for bacterial expression (Fig 2). A 6-His tag was inserted in the C-terminal end of one of the trx molecules for purification on nickel-nitrilotriacetic acid (Ni-NTA) agarose beads (Qiagen, GmbH, Hilden, Germany). In the linker region between the two-trx molecules, the different substrate sequences were inserted by ligating double-stranded oligonucleotides into two unique restriction sites, *BamHI* and *SalI*. The oligonucleotides for the minimal sites were ordered from Sigma-Aldrich. The longer constructs encoding the cleavage sites including also N- or N+C-terminal regions were all ordered from Genscript Corporation (USA). Correct inserts were confirmed by restriction enzyme digestion of the plasmid mini-preps made from the overnight cultures grown at 37°C (E.Z.N.A. kit from Omega Bio-Tek, Doraville, USA and according to the manufacturer’s protocol). After cloning, the individual clones were verified by sequencing of both DNA strands.

The recombinant pET21-trx plasmids were then transformed into the *E*.*coli* Rosetta gami strain for protein expression (Novagen, Merck, Darmstadt, Germany). Cells were spread on LA-ampicillin (amp) plates and incubated at 37°C overnight. One distinct colony was picked and grown overnight at 37°C in 10 ml LB containing 50 μg/ml of amp. The 10 ml overnight culture was added to 90 ml LB containing 50 μg/ml amp and 0.1% glucose. Bacteria were grown at 37°C with shaking for approximately 1 hour until an OD600 value reached 0.5. Isopropyl β-D-1-thiogalactopyranoside (IPTG) was added to a final concentration of 1mM to induce protein expression. The cells were then incubated for an additional 3 hours at 37°C under vigorous shaking. The cells were harvested by centrifugation at 4°C for 10 minutes at 6,000 rpm. The cell pellet after IPTG induction was re-suspended in 25 ml PBS containing 0.05% Tween and subsequently spun down for 10 minutes at 6,000 rpm and 4°C. The supernatant was removed and pellet re-suspended in 2 ml PBS (1/50th of starting volume). Cells were lysed by sonication in the intervals of 5 x 30 seconds (30 seconds sonication followed by a cooling step on ice for 30 seconds). The cells were centrifuged for 10 minutes at 10,000 rpm and 4°C after each washing step. The supernatant was removed and 0.5ml PBS containing 0.05% Tween was added. The tube was sonicated in short bursts (1 or 2 seconds) to resuspend the pellet. Finally the resuspended cell pellet was centrifuged at 10,000 rpm for 5 minutes at 4°C and the supernatant was transferred to a separate tube.

To purify the protein, the pooled supernatants containing the protein was mixed with 250 μl Ni-NTA agarose (Qiagen, Gmbh, Hilden, Germany) (roughly 125 μl beads) and were left to rotate at room temperature for 1 hour. Following brief centrifugation for approximately one minute at 3000 rpm and 4°C, the supernatant was removed and the beads were transferred to a 1.5 ml microcentrifuge tube. The beads were washed 4 times with 1 ml PBS + Tween 0.05%. The tube inverted several times to mix thoroughly then the beads were briefly centrifuged for 20 seconds to allow removal of the supernatant. To elute the protein, the beads were transferred to a 2 ml syringe (Terumo Europe, Leuven, Belgium) containing a glass filter (Whatman, England). The beads were washed in three steps, first 1 ml, then 2 ml and a final 2 ml wash. All three washes were performed with PBS + 0.05% Tween and 20 mM imidazole. Bound protein was eluted in 6 fractions: 1st 100 μl, 2nd to 6th 200 μl with PBS + 0.05% Tween containing 100 mM imidazole. For some proteins the imidazole concentration had to be increased to 300 mM to elute the protein for unknown reasons. Each fraction was collected individually and a small aliquot of the eluted protein was mixed with sample buffer, β-mercaptoethanol and then heated for 2 minutes at 90°C. Eluted protein was analyzed by SDS-polyacrylamide gel electrophoresis (SDS-PAGE) on 4–12% gradient SDS PAGE gels (Novex life technologies, Invitrogen, CA) and the gel was stained overnight with colloidal Coomassie blue [30]. The fractions containing the most protein were pooled and the protein concentration of the combined fractions was determined using the Bradford protein assay [31].

### Recombinant substrate cleavage by thrombin

Approximately 60 μg of recombinant protein was added to each 75 μl cleavage reaction (in PBS). Fifteen μl from this tube were removed before adding thrombin for the 0 minute time point. Thrombin was then added to a final concentration of 1 nM and the reaction was kept at room temperature during the entire experiment. Fifteen μl from this tube were removed at the indicated time points (15, 45 and 150 minutes) and the reactions were stopped by the addition of 5 μl of 4X sample buffer. One μl β-mercaptoethanol was added to each sample followed by heating for 3 minutes at 90°C. The entire sample of 20 μl from each of the time points were analyzed on 4–12% pre-cast SDS Bis-Tris PAGE gels (Invitrogen, Carlsbad, CA, USA). The gels were stained overnight in colloidal Coomassie staining solution and de-stained between 4–5 hours to over night [30]. All protein gels were analyzed using Image Quant TL 1D gel density software (v8.1) from GE Life Science (Piscataway, NJ USA) or the UN-SCAN-IT Gel Analysis Software from Silk Scientific Inc. (Orem, Utah USA).

Cleavage reactions with protein C was also performed with thrombomodulin in the presence of Ca2+ ions and with or without polyethylene glycol (PEG). The reaction volumes for the recombinant substrates were as described above. In the reaction mixes thrombomodulin was added to a molar concentration of approximately 10 times that of thrombin to ensure sufficient number of thrombomodulin molecules to be present in the reaction mix to allow efficient activation of protein C substrates. The reaction conditions were also tested for the activation of native non-activated protein C by cleavage of a chromogenic substrate with preference for active protein C, S-2366. In 200 ul reaction volumes with final concentrations of 10 mM Tris pH 7.5, 2.5 mM CaCl2 0.2 mM S2366, Thrombin 4ng/ 200 ul, thrombomodulin 51 ng/200 ul, protein C 0.69 ug/200ul.

## Results

### Construction of recombinant substrates

A quantitative analysis of the involvement of exosites for the cleavage of different activation sites by human thrombin has previously been technically quite challenging. The only possible ways have been the production of mutated variants of recombinant proteins or by long synthetic peptides, which has made it difficult to make a more broad analysis of a large number of important sites. To solve this problem we have developed a new type of recombinant protein substrate based on a cleavable site between two trx proteins. Previous results have shown highly reliable and accurate quantitative estimates on the importance of individual amino acid positions in substrate cleavage by the human mast cell chymase, the dog mast cell chymase, thrombin, the macaque chymase and a number of other enzymes not yet published [28, 32–34]. Today, more than 120 different substrates have been produced and during their cleavage analysis with a large panel of enzymes, no indications for interactions between the enzyme and the trx molecules have been observed.

**Fig 4:**
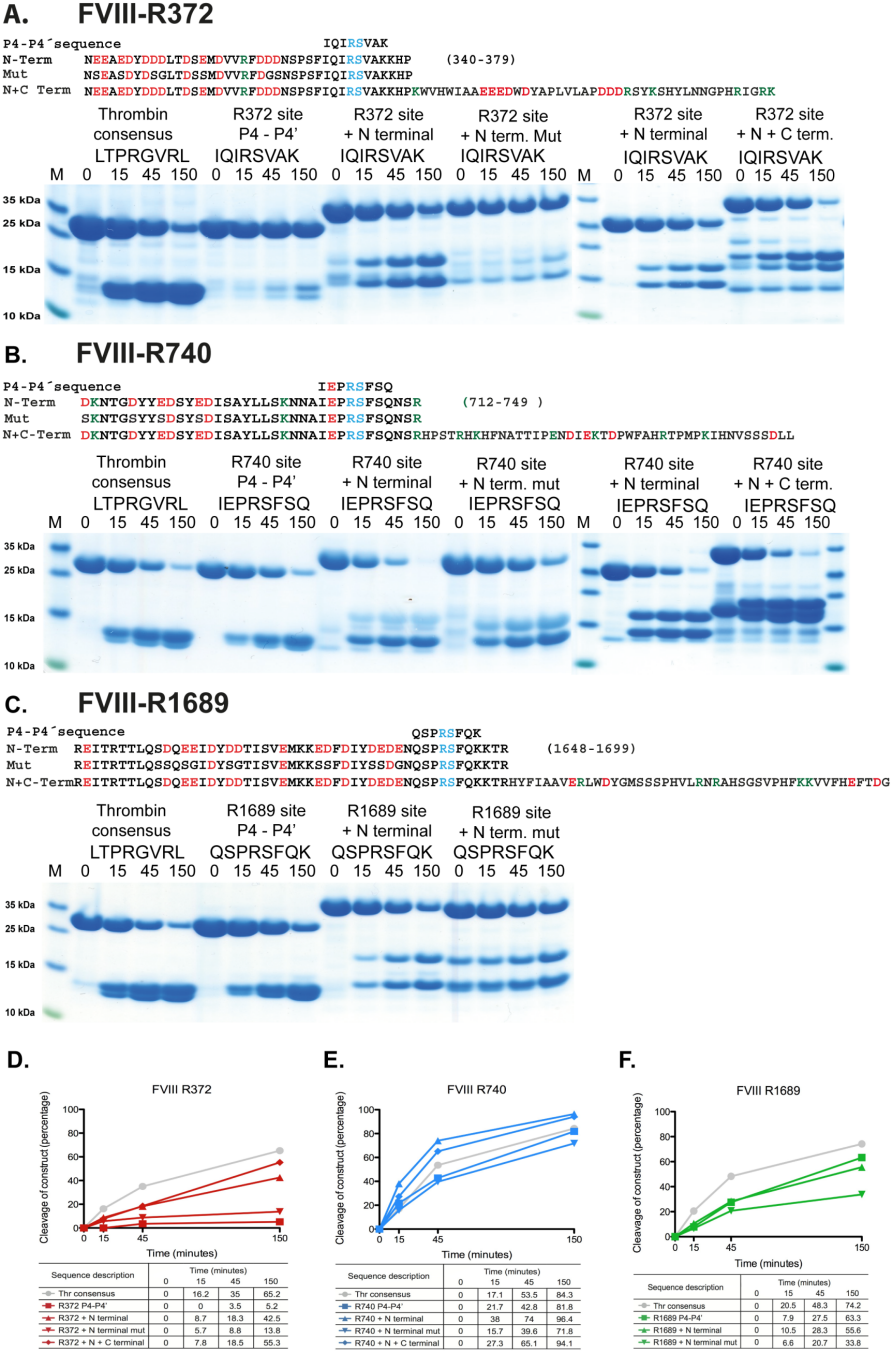
The importance of exosite interactions for the cleavage efficiency of cleavage sites in FVIII. The name and sequence of the substrates are indicated above the gel pictures. The time of cleavage (in minutes) is also indicated above their corresponding lanes on the gel. Panels A-C shows the results for the individual cleavage sites in FVIII, R372, R740 and R1689, respectively. Panels D, E and F shows the results from a scanning of the individual gels with corresponding percentages for a more easy evaluation of the result.

**Fig 5:**
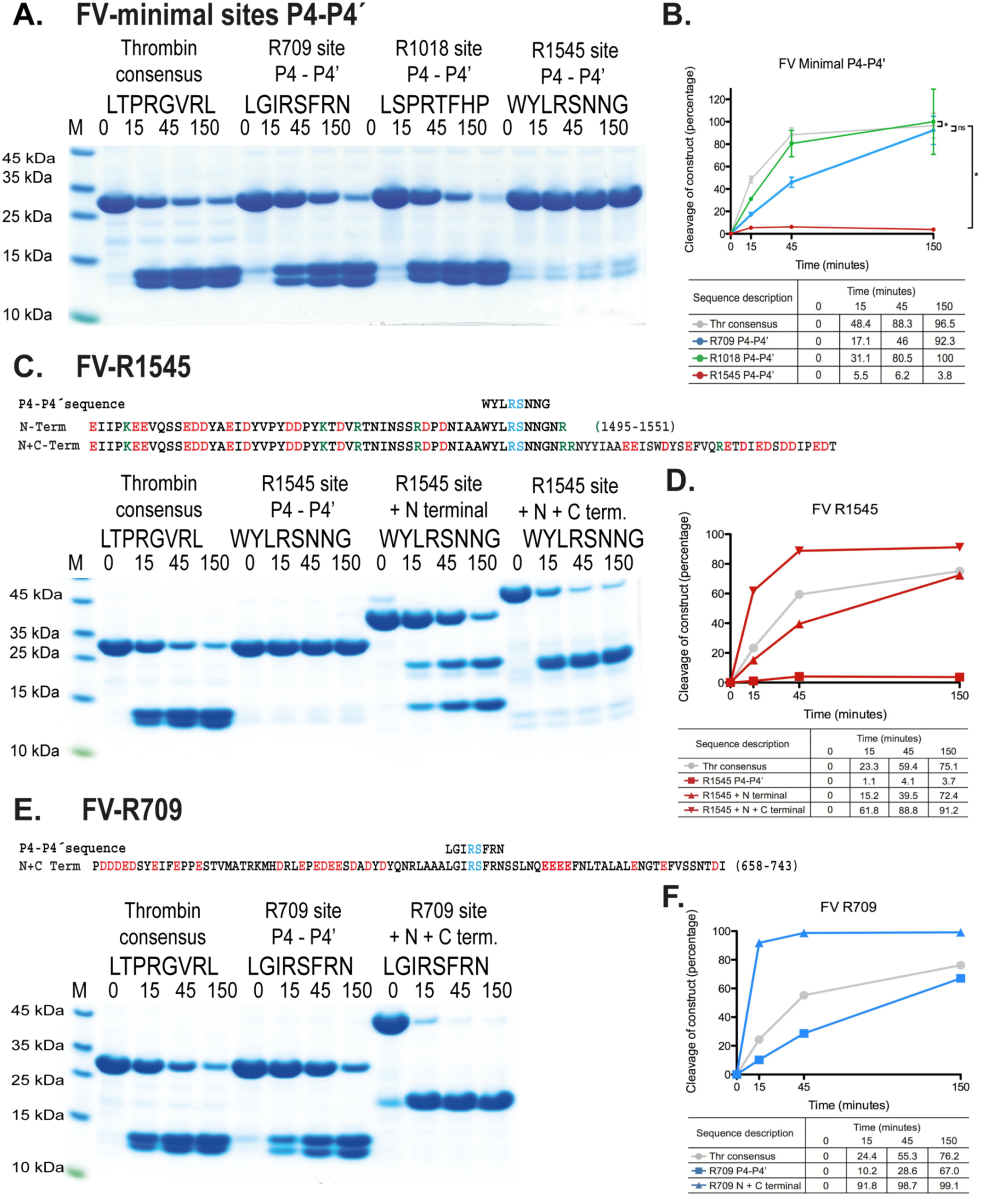
The importance of exosite interactions for the cleavage efficiency of cleavage sites in FV. The name and sequence of the substrates are indicated above the gel pictures. The time of cleavage (in minutes) is also indicated above their corresponding lanes on the gel. Panels A shows the results from the analysis of the minimal sites for FV, R709, R1018 and R 1545. Panels B, D and F shows the results from a scanning of the individual gels with corresponding percentages for a more easy evaluation of the result. Standard deviation of the time points are shown (mean +- standard deviation). Statistical analyses were performed using the Mann-Whitney test with two-tailed P value. **p value = 0.0079, *p value = 0.0119, ns, not significant.

**Fig 6:**
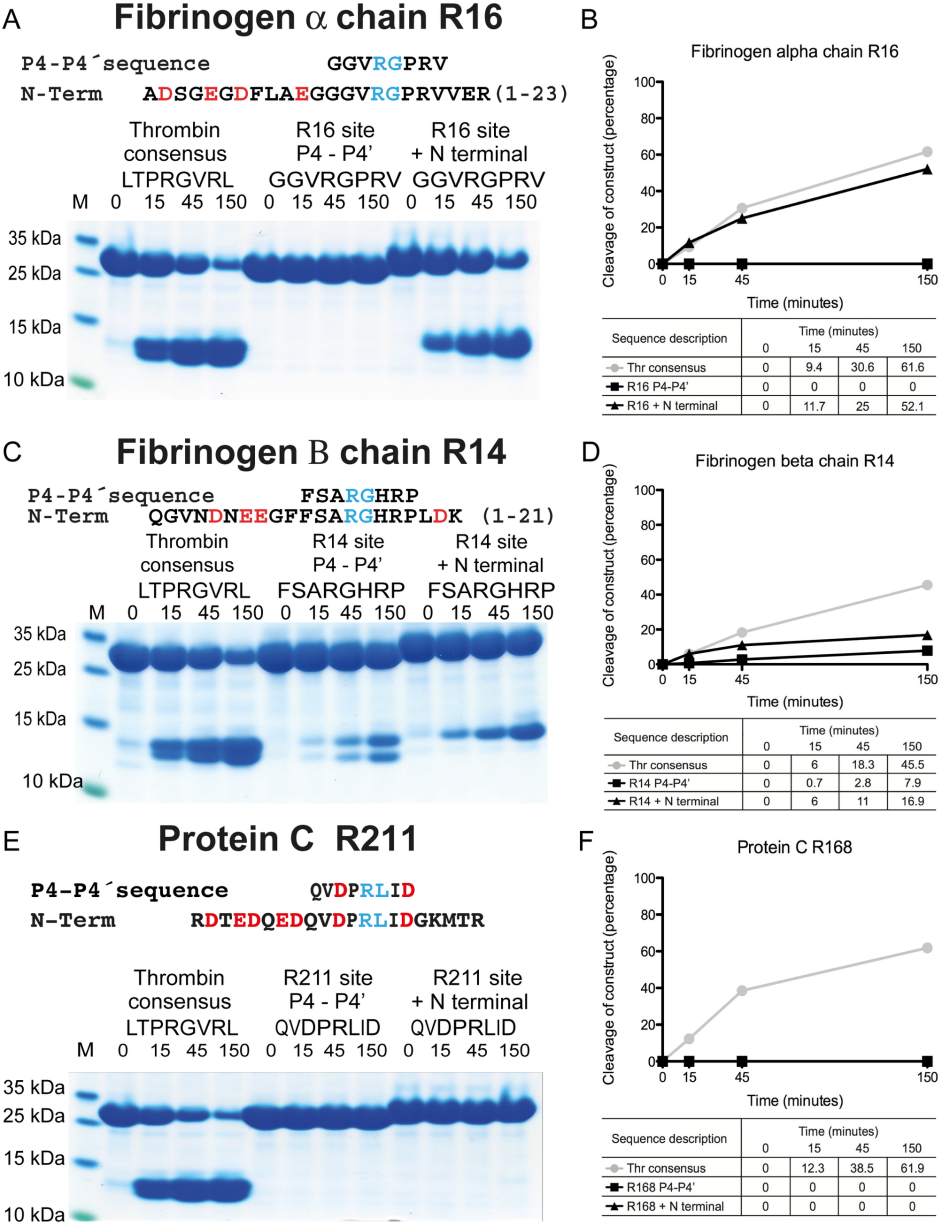
The importance of exosite interactions for the cleavage efficiency of cleavage sites in fibrinogen α chain (panel A), β chain (panel C) and protein C (panel E). The name and sequence of the substrates are indicated above the gel pictures. The time of cleavage (in minutes) is also indicated above their corresponding lanes on the gel. Panels B, D and F shows the results from a scanning of the individual gels with corresponding percentages for a more easy evaluation of the result.

**Fig 7:**
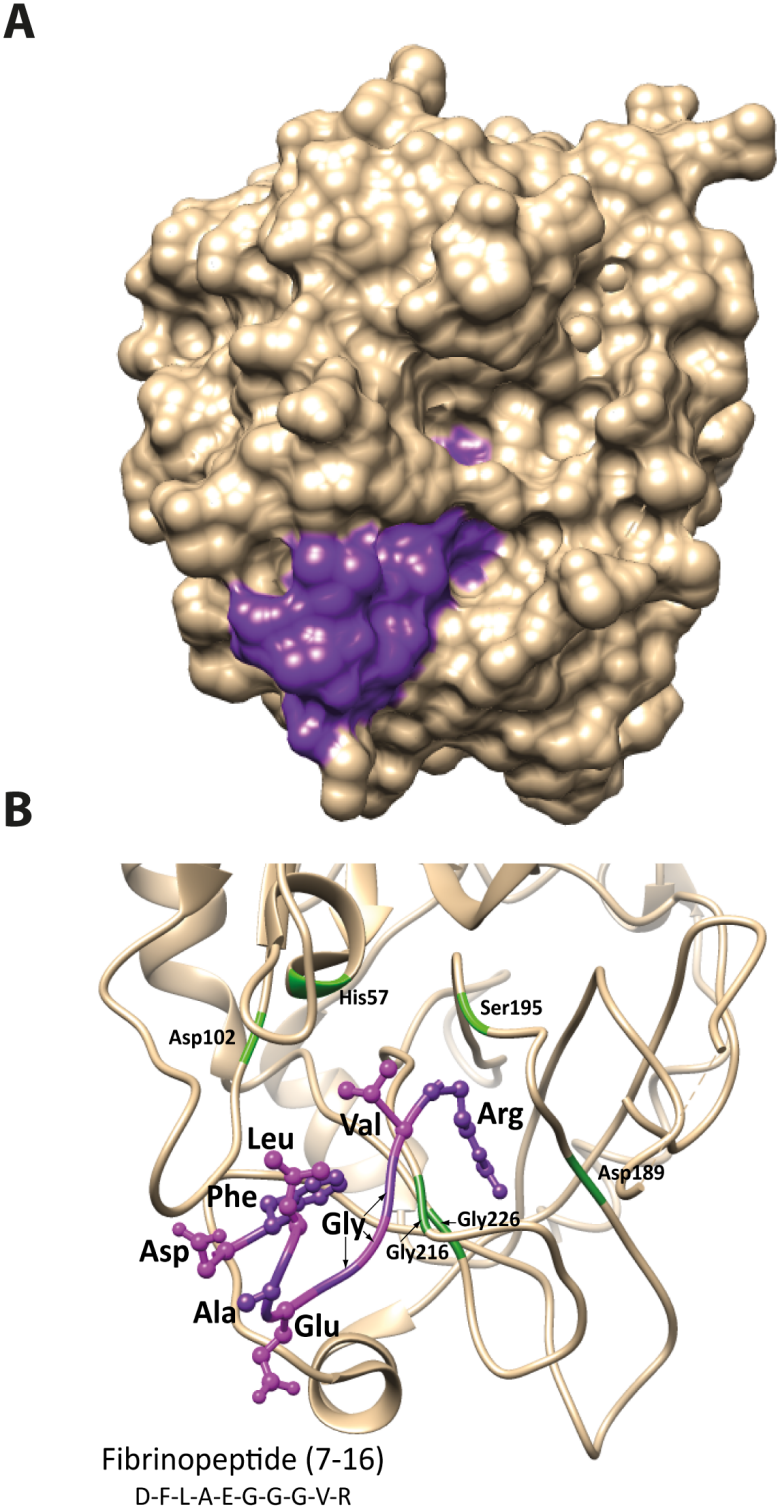
Schematic 3-D models of human thrombin showing the position of the N-terminal region of fibrinogen α chain. Panel A shows a space-filling model with the alpha chain peptide in purple. Panel B shows the interaction between thrombin (ribbon structure in beige) and the N-terminal region of fibrinogen α chain (ball and stick structure in purple) in detail. The same orientation as panel A is shown with the catalytic residues His57, Asp102 and Ser195 together with the S1 pocket residues Asp 189, Gly216 and 226 in green. Thrombin structure from PDB, code 1DM4 run using UCSF Chimera v1.8 and annotated in Adobe Illustrator CS5.

**Fig 8:**
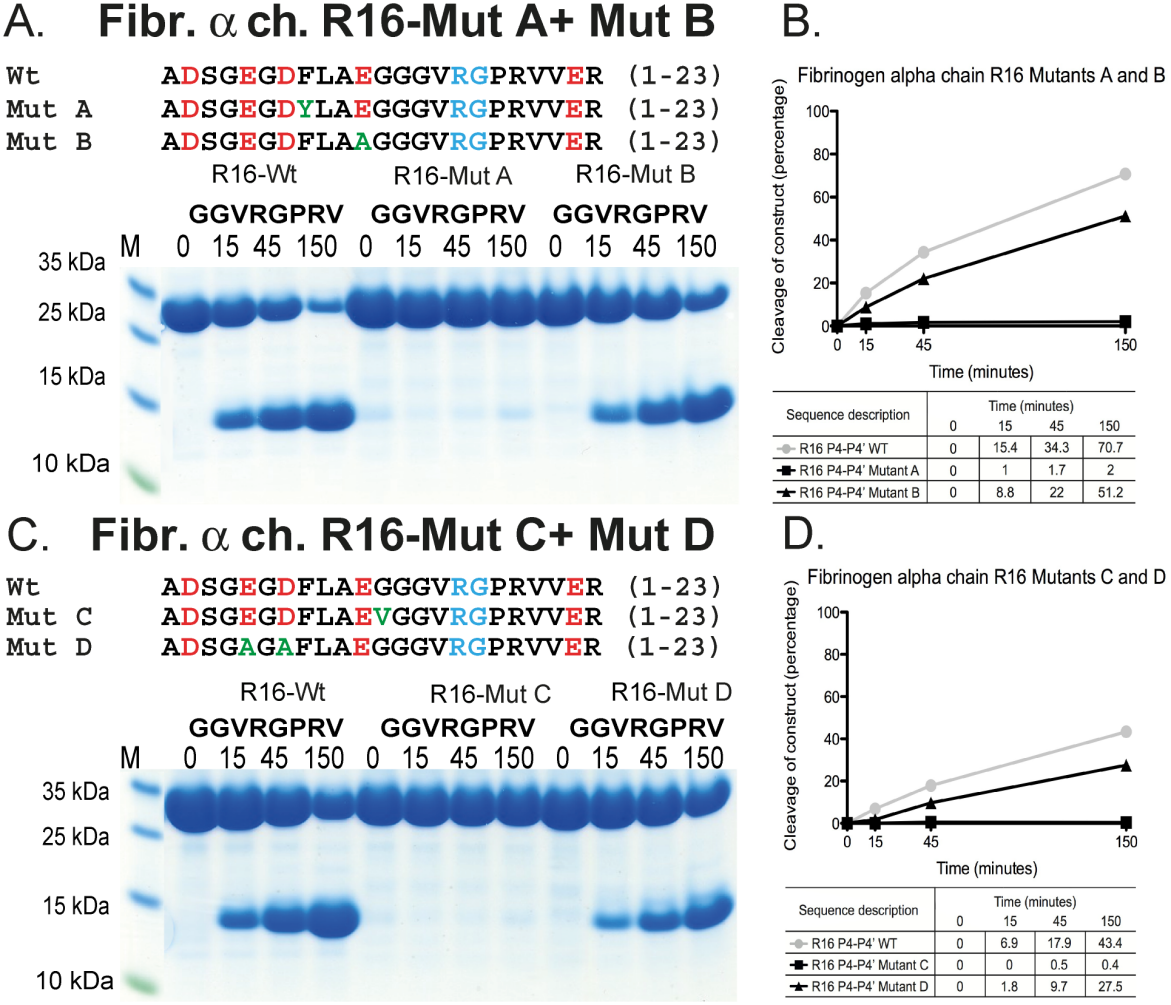
Analyses of the importance of Phe8, Gly12 and three negatively charged residues in the N-terminal region of fibrinogen α chain for the cleavage by thrombin. The name and sequence of the substrates are indicated above the gel pictures. The time of cleavage (in minutes) is also indicated above their corresponding lanes on the gel. The mutations are marked in green in the sequences above each gel. Panels B and D shows the results from a scanning of the individual gels with corresponding percentages for a more easy evaluation of the result.

To study the involvement of the exosites for a number of important activation sites for human thrombin, we designed and ordered complementary single-stranded oligonucleotides encoding the region covering the thrombin cleavage sites with four amino acids up- and downstream of the cleavage sites in human FV, FVIII, fibrinogen α chain, fibrinogen β chain and protein C (Fig 3). This region is named the P4-P4’region or the minimal site. After mixing the paired oligonucleotides they form a stable double-stranded oligonucleotide with BamHI and SalI sticky ends. These were cloned into the Bam HI and SalI sites of a cleaved bacterial expression, pET21-2x trx, vector. This vector contains two *E*. *coli* trx genes with a linker region in between where different sequences can be introduced followed by a downstream six-His tag. (Fig 3). For the longer sequences also covering the negatively charged regions N-terminal, or both N- and C-terminal of the cleavage site, these regions were ordered as designer genes, cloned and sequence-verified from Genscript. In total, 28 different constructs were produced: FVIII-Arg372 minimal, FVIII-Arg372 N-region, FVIII-Arg372 N-region mutated, FVIII-Arg372 N+C-regions, FVIII-Arg740 minimal, FVIII-Arg740 N-region, FVIII-Arg740 N-region mutated, FVIII-Arg740 N+C-regions, FVIII-Arg1689 minimal, FVIII-Arg1689 N-region, FVIII-Arg1689 N-region mutated, FVIII-Arg1689 N+C-regions, FV-Arg709 minimal, FV-Arg709 N+C-regions, FV-Arg1018 minimal, FV-Arg1018 N+C-regions, FV-Arg1545 minimal, FV-Arg1545 N-region, FV-Arg1545 N+C-regions, fibrinogen α chain-Arg16 minimal, fibrinogen α chain-Arg16 N-region, fibrinogen β chain minimal, fibrinogen β chain N-region, protein C-Arg168 minimal and protein C-Arg168 N-region. The sequences of all the individual inserts are shown above each SDS-PAGE gel with the cleavage reaction for FVIII, FV, fibrinogen α chain, fibrinogen β chain and protein C respectively (as shown in Figs 3–6). Four different mutated variants of the N-terminal region of the fibrinogen α chain-Arg16 were also produced to study the importance of individual amino acid positions in this region of the target molecule (Figs 7 and 8).

In addition to the described clones, we also used the human thrombin consensus cleavage site, the most optimal cleavage site determined by phage display (LTPRGVRL) as a reference (Figs 3–6) [28].

Following the cloning and verification of the insert sequences, all clones were transferred to the *E*.*coli* Rosetta gami strain for efficient expression of recombinant proteins. After IPTG induction and cell sonication to open up the cells, a single step purification on Ni-chelating IMAC columns resulted in more than 90% pure protein ready for the use in the cleavage reactions (Fig 3).

Each set of results per SDS-PAGE gel shown were run independently, with the thrombin consensus control run 10 times in total with similar results, further highlighting the robustness of the system. Gel density readings of the cleaved constructs were also calculated using Image Quant TL 1-D gel software (GE Life Science) (the scanning results are shown as separate panels in Figs 3–6 and 8).

### The cleavage sites Arg372, Arg740 and Arg1689 in human coagulation factor VIII

To determine the cleavage efficiency of thrombin for the three cleavage sites in FVIII, approximately 60 μg of the recombinant substrate encoding the minimal sites were subjected to cleavage by thrombin. Samples were taken after 0, 15, 45 and 150 minutes of digestion and analyzed by SDS-PAGE. The cleavage efficiency of these minimal sites was compared with the optimal sequence for human thrombin (LTPR↓GVRL, where the arrow indicates cleavage). The results showed that thrombin cleaves the Arg740 and the Arg1689 sites almost as efficiently as the thrombin consensus sequence (Fig 3). However, the Arg372 site showed almost no cleavage under the same conditions (Fig 3). To determine the reproducibility of the assay the samples for the minimal sites were run 5 times and scanned and the standard deviation was determined (S1 Fig). As seen from S1 Fig, Figs 3D and 5B the assays are highly reproducible. The standard deviation is not higher than by using spectrophotometric measurements and chromogenic substrates, but with substrates that is much more biologically relevant. However, to obtain a good estimate of the relative difference in activity multiple runs of the same material is of little value. A much more fruitful approach is to use the same cleavage material but instead run these samples with different amounts of enzyme to get a detailed estimate of the difference in cleavage activity. So by using varying amounts of thrombin we estimated the difference in cleavage rates between the substrates (Fig 3 and data not shown). The amount of cleavage observed for Arg372 indicated the site was approximately 30–40 times less efficient as compared to the thrombin consensus sequence.

### Determining the importance of exosite interactions for the cleavage at the three cleavage sites in FVIII

To determine the role of the negatively charged regions located upstream of the cleavage sites, the clones containing the N-terminal region, and the N- and C-terminal regions were analyzed by *in vitro* cleavage.

The addition of an approximately 30 amino acid region N-terminal of the Arg372 cleavage site resulted in a major increase in cleavage efficiency (Fig 4). The region covering amino acids 340–379 of human FVIII was cleaved almost as efficiently as the thrombin consensus sequence. This region contains 13 negatively charged amino acids (in bold, with the cleavage site indicated by an arrow) (340–379:N**EE**A**ED**Y**DDD**LT**D**S**E**M**D**VVRF**DDD**NSPSFIQIR↓SVAK-KHP) (Fig 4). To analyze the importance of the negatively charged amino acids for this increase in cleavage efficiency, approximately 50% of them were mutated into glycine or serine. The selection of which residues to mutate was decided on the basis of getting a relatively even distribution of the reduction in charge density over the entire region. Therefore, approximately every second negatively charged residue was mutated. The reduction in charge density by these mutations resulted in a cleavage rate almost identical to the minimal site, suggesting that a high negative charge is of major importance for the enhancement in cleavage rate (Fig 4). Addition of both N- and C-terminal sequences to the Arg372 site resulted in small additional increase in cleavage efficiency, approximately 1.5–2 fold, indicating that both N- and C-terminal sequences are involved, but that the C-terminal region for this site is of minor importance.

A cluster of negative charges (in bold) is also found N-terminal to the Arg740 site (712–749: **D**KNTG**D**YY**ED**SY**ED**ISAYLLSKNNAI**E**PR↓SFSQNSR). Seven acidic residues are found within a region of approximately 35 amino acids N-terminal of this site. When this region was included in the recombinant substrate, thrombin cleaved this sequence 1.5–2 times more efficiently than the minimal site. A complete loss of this enhancing effect was seen after replacing 4 of the 7 negatively charged residues with serine or glycine (Fig 4). This result showed that exosite interactions are also of importance for the Arg740 site, although to a much lesser extent. Addition of both N- and C-terminal sequences did not result in any additional enhancement over the N-terminal sequence alone (Fig 4).

A high number of acidic residues are also found N-terminal of the Arg1689 cleavage site. Within a region of approximately 40 amino acids, 15 negatively charged amino acids (in bold) and two potentially sulfated tyrosines are found (1648–1696: R**E**ITRTTLQS**D**Q**EE**I**D**Y**DD**TISV**E**MKK**ED**F**D**IY**DEDE**NQSPR↓SFQKKTR) (Figs 1 and 4). When this region was included in the recombinant substrate no enhancement of the cleavage rate over the minimal site was observed. However, mutations within this region resulted in a marked reduction of the cleavage rate over the minimal site (Fig 4).

### The cleavage sites Arg709, Arg1018 and Arg1545 in human coagulation factor V

The minimal sites for the three cleavage sites for thrombin in human FV were analyzed by *in vitro* cleavage. The Arg709 and Arg1018 sites were found to be almost as good sites as the consensus sequence, whereas site Arg1545 showed almost no cleavage under the same conditions (Fig 5).

By adding the N-terminal region to the Arg1545 site, which contains 13 acidic amino acids within a region of 45–50 amino acids, the cleavage efficiency reached that of the consensus site (Fig 5). Addition of both N- and C-terminal regions resulted in a cleavage that was 2–3 times better than the consensus site, and almost 6 times better than with only the N-terminal region. Here, the C-terminal region of 35–40 amino acids contains 12 negatively charged amino acids. The enhancement with both N- and C-terminal regions for this site was around 50–60 fold (Fig 5).

The minimal site for Arg709 in FV was cleaved almost as well as the consensus site, and addition of N- and C-terminal regions resulted in an enhancement of 6–10 times. Here, the N-terminal region contains 17 negatively charged amino acids within a region of 35–40 amino acids, and the C-terminal region contains 7 negatively charged amino acids in a 30–35 amino acid region (Fig 5).

Site Arg1018 could not be analyzed in more detail as the clone containing both N- and C-terminal regions showed extensive cleavage after expression and purification from the bacteria, most likely by bacterial proteases. This phenomenon was also observed for FVIII clones Arg1689 N-terminal mutant and Arg372 N- and C-terminal regions although to a lesser extent (Fig 4).

### The cleavage sites Arg16 in fibrinogen α chain, Arg14 in fibrinogen β chain and Arg211 in protein C

No cleavage was observed for the fibrinogen α chain Arg16 minimal site even after 150 minutes, which showed that Arg16 is a very poor site for thrombin. However, after addition of only 12 N-terminal amino acids, containing 4 negatively charged amino acids, the cleavage was almost as good as the consensus site, highlighting the very potent effect of exosite interactions (Fig 6A). Using increased amounts of thrombin in the cleavage reaction indicated that the enhancement by the N-terminal region was above 1000 fold (data not shown). The Arg14 in fibrinogen β chain was a relatively efficient substrate for thrombin showing a cleavage of approximately 15% of the optimal site. H, there was a modest enhancement observed with the addition of the 10 remaining amino acids of the N-terminal region to approximately 30% of the optimal site. (Fig 6B).

No cleavage was observed for the minimal site for protein C Arg211 (Fig 6C). However, and in sharp contrast to the fibrinogen α chain, absolutely no cleavage was seen after addition of 8 N-terminal amino acids containing 5 negatively charged amino acids, indicating that other mechanisms apart from the classical exosite interaction are of importance for the cleavage of protein C (Fig 6C). Further addition of thrombomodulin and calcium to the cleavage reaction, with or without polyethylene glycol, for both the minimal site and the substrate containing additional upstream and downstream sequences, did not result in any detectable cleavage from two independent experiments (data not shown). In order to confirm that the conditions were correct we tested a chromogenic substrate for protein C, S-2366 with non-activated protein C, activated protein C in the presence of thrombomodulin, activation in the absence of thrombomodulin and the activity by thrombin alone. All the reactions were performed with calcium and with or without polyethylene glycol. No activity was seen with non-activated protein C or by only adding thrombin (data not sahown). However when thrombomodulin was added the reaction was rapid and strong (data not shown). These results show that the conditions for cleavage of the recombinant substrates were optimal for protein C activation. However, no cleavage was observed under these conditions of any of the protein C recombinant substrates, the minimal site and the N-terminal region (Fig 6).

### Mutational analyses of the N-terminal region of the cleavage site Arg16 in fibrinogen α chain

The N-terminal region of the fibrinogen α chain has previously been studied in relative detail by crystallographic studies and by mutational analysis (Fig 7). The crystallographic analyses show that the N-terminal region of the fibrinogen α chain bind close to the active site where Phe8 and Gly12 are in tight contact with thrombin (Fig 7) [35]. Mutation of Phe8 into a highly similar amino acid Tyr and Gly12 into a Val has previously been shown to have a major inhibitory effect on cleavage [35]. To verify these studies and to study the importance of the negatively charged residues in this region, we constructed four different mutants and analyzed them against the wild-type sequence for their cleavage rate against human thrombin. The results from Binnie and Lord 1993, were accurately verified using the two trx approach, highlighting the major importance for cleavage activity by Phe8 and Gly12 [35]. These two mutations each resulted in a drop in cleavage activity by several hundred fold (Fig 8). We also made two additional mutants, one by replacing Glu11 with Ala and another where Glu5 and Asp7 were also replaced with alanines (Fig 8). Both of these mutant variants had little effect on the cleavage activity, clearly demonstrating the extreme selectivity between very closely positioned mutations on the substrate cleavage in this region of the molecule.

## Discussion

A new type of recombinant substrate has been used to study the involvement of exosite interactions, and also to obtain semi-quantitative information concerning the importance of such interactions for nine important cleavage sites for thrombin. This new tool has made it possible to answer questions that have been very difficult to previously address. To make large numbers of mutations in recombinant FVIII, FV, fibrinogen α and β chains, and protein C is very time consuming and costly, which is further compounded as some proteins are very difficult to express. This is particularly apparent with the very complex proteins analyzed in this communication. It is almost impossible to obtain quantitative measurements with such large proteins. Synthetic peptides can be used but are not without their own problems related to accuracy in synthesis and very high cost when producing long peptides. Here we have analyzed regions of approximately 100 amino acids in length and such long peptides are very costly, to make synthetically. The analysis of such peptides by MS is also not fully quantitative. Therefore, to solve these problems we developed a system that could address most of these issues and make it possible to address general questions concerning exosite dependence. Here we have analyzed eight different activating sites and one negatively regulating cleavage site. The presentation of the exosite regions in a non-conformation-dependent manner has also made it possible to at least partly separate the influence of charge from conformation-dependent factors. The analyses showed that exosite interactions are primarily of importance for poor sites for thrombin as exemplified by FVIII-Arg372, FV-Arg1545 and fibrinogen α chain-Arg16, where the increase in cleavage efficiency can exceed 1000 fold with the addition of the exosite interacting regions of the substrate. Minimal sites that are almost as efficiently cleaved as the consensus site are comparably less enhanced by the addition of such regions. In some cases, as with FVIII Arg1689, no enhancement was observed and with FVIII Arg740, only a 2–3 fold enhancement was detected. However, a more pronounced effect could also be seen, for example in FV-Arg709 where a 6–10 fold increase in cleavage with the addition of the N- and C-terminal regions was seen (Figs 4 and 5).

By mutating approximately half of the negative charges in the three FVIII sites, the enhancement was lost almost completely. This latter finding clearly shows the importance of the electrostatic interaction for the enhancement of at least these sites in FVIII. However, as discussed above, the effect of these mutations was most prominent on the poor FVIII sites (Fig 4). The question then arises as to why strongly negatively charged regions are present at all of these sites in both FV and FVIII, except FV-Arg1018, when they seem to be of importance only, or at least primarily, for the poor sites (Fig 1). Do they also have another function or are they reminiscent of an internal duplication process generating the three sites? A third possibility is that they are of importance to keep the cleavage sites accessible, as charges are often exposed on the surface of a protein. Crystallographic studies of FVIII has shown that these negatively charged regions show little if any ordered structure, indicating they are flexible and open for protease attack, which favors the third alternative [36]. This also makes our analyses more biologically relevant as our recombinant substrates most likely have a similar unordered structure as in the natural substrates.

Mutational analysis of hirudin, a thrombin inhibitor that interacts with ABE-I, shows a remarkably small effect on binding affinity of individual mutations, which indicates that the sum of the interactions is what is important also for the formation of the thrombin-hirudin complex [37, 38].

Studies of recombinant FVIII have shown that several of the tyrosines are sulfated, increasing the negative charge of these regions. Interestingly, addition of sulfate groups on tyrosine in FVIII seems almost exclusively to occur on tyrosines that border the thrombin cleavage sites and thereby are found in the structurally open regions [39]. Addition of sodium chlorate to the culture medium, which blocks sulfation, results in secretion of FVIII lacking sulfated tyrosines. A five-fold reduction in procoagulant activity is observed with this protein, showing the importance of these additional negative charges for the cleavage by thrombin [39]. However, a partial lack of sulfate groups did not seem to affect *in vitro* clotting activity [40]. Interestingly, when we look at the individual sites in FVIII, the majority of these tyrosines are located upstream of the two good cleavage sites Arg740 and Arg1689, with four and two tyrosines, respectively (Fig 4). In these positions we would expect them to have a relatively limited effect on the cleavage efficiency as these sites are already very good. However, when we look both upstream and downstream of the site, all of them have approximately the same number of tyrosines, Arg372 has one upstream and four downstream of the cleavage site, Arg740 has four upstream and no downstream and Arg1689 has two upstream and two downstream of the cleavage site. The accumulated effect of these sulfated tyrosines probably gives the five-fold difference in coagulation activity [39]. Although, the joint effect of all three together may be quite substantial, the individual effect of these sulfated tyrosines is probably difficult to observe in our assay.

Due to the relatively large regions with a high percentage of negative charge N-terminally and sometimes both N- and C-terminally of the cleavage site, the interaction with both ABE-I and -II on thrombin would theoretically be possible. This assumption is based on the distance between the active site and the ABE-I and-II (Figs 1 and 2). The additional enhancement when adding C-terminal sequences (as shown in Figs 4 and 5) indicates that both exosites are involved. However, for both the fibrinogen α and β chains, the potential exosite-interacting region is short and does not extend far enough from the active site to reach ABE-I or-II. The crystal structure of thrombin bound to the N-terminal region of fibrinogen α chain has been solved, showing that this N-terminal region interacts with a region close to the active site of thrombin, not involving ABE-I or–II (Fig 7) [35]. The importance of a few non-charged amino acids in the fibrinogen α chain propeptide and a region close to the active site of thrombin in this article has been beautifully demonstrated [35]. Interestingly, a Phe and a Gly were found to be essential for this interaction, also showing that other sites apart from ABE-I and-II can be of major importance and that electrostatic interactions are not always key components in this interaction. The Phe is most likely forming a strong hydrophobic interaction that is so specific that not even a very closely related amino acid, Tyr, can replace it [35]. Interestingly, a Gly was also found to be very important and the effect is probably related to bending of the peptide chain, facilitating other amino acids to find their proper interacting environment [35]. Using the recombinant trx substrates we have been able to confirm these studies and obtain quantitative estimates of the importance of these amino acid positions as well as additional information concerning the importance of charged residues in this region. In our system, both the Phe8 and the Gly12 mutations reduced the cleavage efficiency by several hundred fold (Fig 8). Making two additional mutants within this region, one involving only one negative charge Glu10 into Ala, and one with two mutations Glu5 and Asp7 into alanines, showed that charge has remarkably little effect, if any, on this interaction. This is in sharp contrast to the poor cleavage sites in FVIII and FV, as discussed earlier. This study also shows the strengths of the recombinant substrates. With this system numerous mutants were analyzed relatively easily, cost effectively, and with high accuracy, with equipment that is available in any biochemistry or molecular biology lab in a matter of weeks. A number of these substrates also contain the entire interacting regions, which is also in sharp contrast to the standard chromogenic substrates, making it is easier to obtain quantitative measurements whilst still mimicking a true biological situation. This is highly relevant for thrombin, which is highly dependent on the extended cleavage site, as shown here (and by many other labs) and on exosite interacting regions [28].

The lack of any effect of the highly charged region surrounding Arg211 in protein C provides another example that electrostatic interactions are not always sufficient for the enhancing effect (Fig 6C). The Arg168 in protein C is an exceptionally poor site for thrombin, due to the Leu and Asp in the P1´and P3´ positions respectively [28]. The major question concerns by what mechanism thrombin or protein C is modified to facilitate cleavage. The activation of protein C by thrombin has been shown to be highly dependent on the interaction with thrombomodulin, and that domains 4, 5 and 6 are essential and sufficient for this interaction [41]. Domains 5 and 6 bind to ABE-I and domain 4 can interact with the region surrounding the active site on thrombin, and is probably also the domain that directly binds protein C [41]. It has been proposed that the interaction between thrombin protein C and thrombomodulin changes the catalytic site of thrombin and thereby its extended specificity [42] [43]. In our hands the addition of thrombomodulin to the cleavage reaction with the protein C minimal site did not result in any detectable cleavage, which indicates that a change in the specificity of thrombin is not a likely explanation. Thrombomodulin has also been proposed to relieve a caging of Arg211 by four negatively charged amino acids of the propeptide [44]. However, the minimal site lacks this region and therefore no caging can occur by these regions, yet we still do not see any cleavage. Both of these two explanations are therefore less likely to give a full explanation for the activation of protein C. Interestingly, one of the sites in prothrombin is also very similar to the site in protein C (RELLESYIDGR^↓^IVEG and TEDQEDQVDPR^↓^LIDG, respectively) indicating similar mechanisms for these to sites for efficient cleavage [44]. Regions at a distance for the activation site in protein C, including the N-terminal γ-carboglutamic acid domain and its coordination of Ca2+, have also been shown to have a strong effect on cleavage, indicating that proper positioning of thrombomodulin, thrombin and protein C in a complex is of major importance for efficient cleavage [45]. Activity of several additional proteases has been shown to be highly dependent on the interaction with other proteins as exemplified by complement factors B, C2 and D and FVII and FXa [44]. It is also possible that two hematopoietic serine protease, the mouse mast cell proteases (mMCP)-2 and the basophil specific serine protease mMCP-8 need cofactors for activity, as they appear to be fully functional with an intact catalytic triad, proper cysteine bridges and expressed at high levels but still no activity has been observed with the pure proteases [29]. The role of cofactor interactions for mMCP-2 may be questionable as this protease has suffered a unique two amino acid deletion in the substrate binding pocked that may be the major reason for its poor activity and not a lack of allosteric regulation [46].

In summary, the minimal sites for two important cleavage sites for thrombin in FVIII and FV were found to be very poor sites for this enzyme. Addition of the highly negatively charged regions upstream of these cleavage sites, increased the cleavage efficiency by 30–60 fold, showing the importance of exosite interactions for poor cleavage sites. Mutants replacing half the negatively charged amino acids with serine or glycine showed the major importance of the negative charge for this enhancing effect. In contrast, when the cleavage sites are close to the optimal for thrombin, the importance of exosite interactions is much less pronounced and sometimes not needed at all. The possibility to mimic the interaction between thrombin and the substrate by a recombinant substrate, which does not have the same folding as the full size target, indicated that this enhancement was primarily dependent on a relatively simple electrostatic interaction. The involvement of exosites other than the classical I and II for fibrinogen α chain and protein C also showed that the regulation of the cleavage by thrombin is very complex and that each situation is resolved individually. However, an underlying question is what the biological significance of these complex sets of regulatory mechanisms is, and if the use of exosites in combination with poor minimal sites enhances the regulatory potential of the system. Of interest is also whether the use of a complex set of interacting surfaces, for example interactions with not only one exosite but two or more, can enhance the fidelity of the system even further. Here, the activation of protein C seems to be exceptionally complex, involving many interactions, which has made deciphering the mechanisms particularly difficult.

## Supporting Information

S1 Fig Statistical analysis of 5 independent experiments with the FVIII and FV minimal sites. The gels from the 10 separate experiments and the scanning results are presented to give a view of the reproducibility of the technique.
(TIF)
