# Supplementary figures and images for: The Importance of Exosite Interactions for Substrate Cleavage by Human Thrombin
Source: PLoS One. 2015 Jun 25;10(6):e0129511. doi: 10.1371/journal.pone.0129511 (PMC4482499; doi:10.1371/journal.pone.0129511)

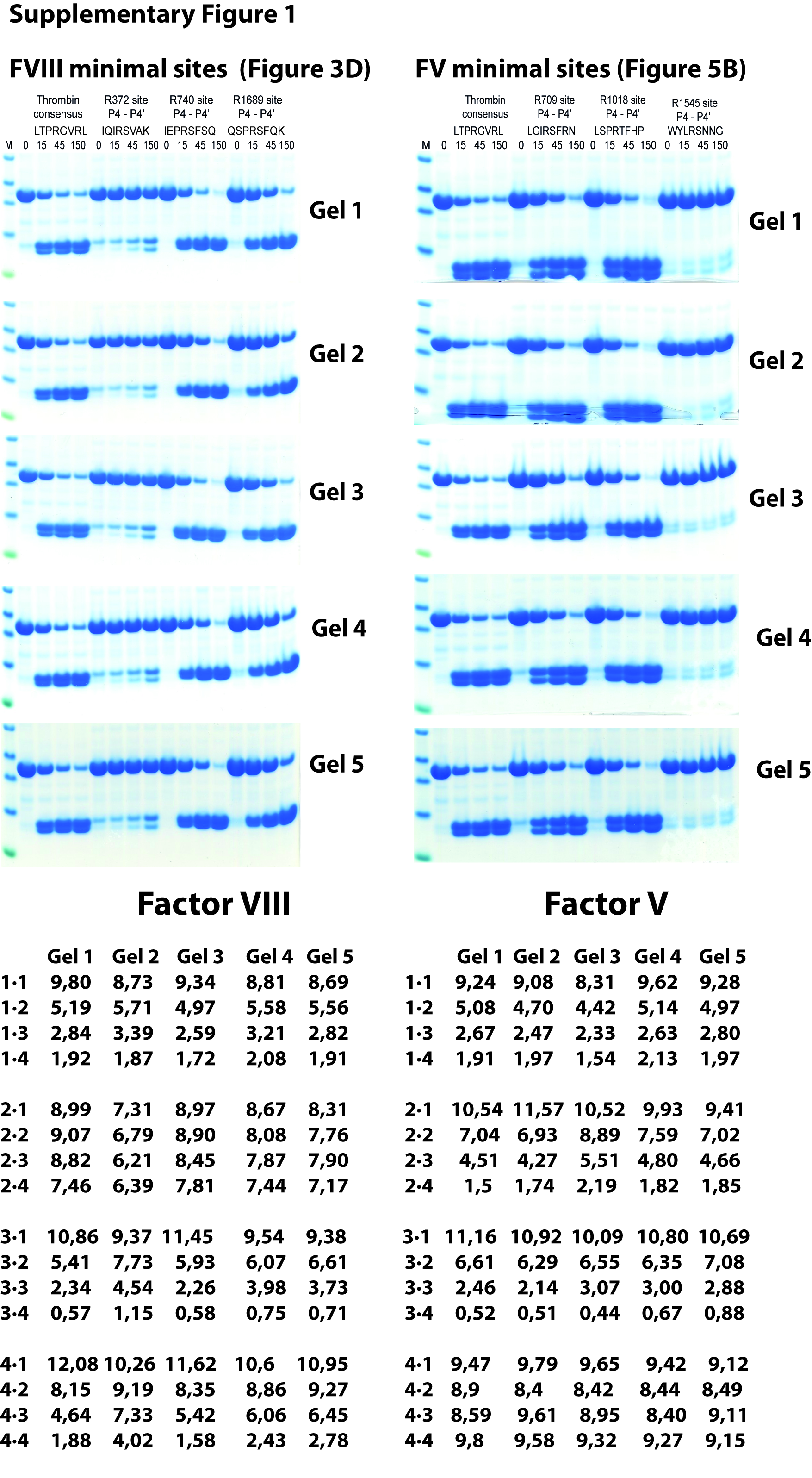

Supplement: S1 Fig — The gels from the 10 separate experiments and the scanning results are presented to give a view of the reproducibility of the technique. (TIF) [file pone.0129511.s001.tif]
